# Exploring the Risk Posed by Animals with an Inconclusive Reaction to the Bovine Tuberculosis Skin Test in England and Wales

**DOI:** 10.3390/vetsci6040097

**Published:** 2019-11-30

**Authors:** Elizabeth May, Alison Prosser, Sara H. Downs, Lucy A. Brunton

**Affiliations:** 1 Veterinary Epidemiology, Economics and Public Health group, Department of Pathobiology and Population Sciences, Royal Veterinary College, Hawkshead Lane, Hatfield AL9 7TA, UK; 2 Data Systems Group, Department of Epidemiological Sciences, Animal and Plant Health Agency, New Haw, Addlestone KT15 3NB, UK; 3 Epidemiology Group, Department of Epidemiological Sciences, Animal and Plant Health Agency, New Haw, Addlestone KT15 3NB, UK

**Keywords:** bovine, tuberculosis, SICCT, inconclusive, reactor, tuberculin

## Abstract

The single intradermal comparative cervical tuberculin (SICCT) test is the primary test for ante-mortem diagnosis of bovine tuberculosis (TB) in England and Wales. When an animal is first classified as an inconclusive reactor (IR) using this test, it is not subject to compulsory slaughter, but it must be isolated from the rest of the herd. To understand the risk posed by these animals, a case-control study was conducted to measure the association between IR status of animals and the odds of them becoming a reactor to the SICCT at a subsequent test. The study included all animals from herds in which only IR animals were found at the first whole herd test in 2012 and used data from subsequent tests up until the end of 2016. Separate mixed-effects logistic regression models were developed to examine the relationship between IR status and subsequent reactor status for each risk area of England and for Wales, adjusting for other explanatory variables. The odds of an animal becoming a subsequent reactor during the study period were greater for IR animals than for negative animals in the high-risk area (odds ratio (OR): 6.85 (5.98–7.86)) and edge area (OR: 8.79 (5.92–13.04)) of England and in Wales (OR: 6.87 (5.75–8.22)). In the low-risk area of England, the odds were 23 times greater, although the confidence interval around this estimate was larger due to the smaller sample size (11–48, *p* < 0.001). These findings support the need to explore differential controls for IR animals to reduce the spread of TB, and they highlight the importance of area-specific policies.

## 1. Introduction

Bovine tuberculosis (TB), caused by *Mycobacterium bovis*, poses a fundamental problem for the cattle industry in both England and Wales. It represents a significant contagious disease, with some areas of England having the highest levels of TB in the European Union [1]. The overall incidence rate in England in 2018 was 11 new cases per 100 herd years at risk. This incidence rate is relatively stable since 2011 [2]. However, there is some evidence of an increase in incidence in the areas surrounding parts of England considered endemic for TB each year between 2015 and 2018 [2,3], suggesting continued spread. In Wales, the incidence rate in 2017 was seven new cases per 100 herd years at risk [3].

Detection of bovine TB in England and Wales is based on routine and risk-based field testing of cattle, as well as post-mortem inspection and tests [4]. Once TB is detected in a herd, trading restrictions are placed on the herd, and animals detected as infected are subject to compulsory slaughter [4]. England and Wales are currently split into geographical risk areas, which determine how regularly cattle are surveillance tested. Cattle within the low-risk area of England, primarily the north and eastern areas, are routinely tested every four years. Those within the high-risk area and the edge area are tested annually; however, 2014 and 2018 saw the introduction of six-monthly testing in herds in some counties in the edge area [4,5,6]. In January 2018, counties that were previously split across the high-risk area (HRA) and edge area were incorporated fully into the edge area [6]. Wales uses annual surveillance testing, as well as twice-yearly testing in a high-incidence part of west Wales known as the intensive action area [7]. In both England and Wales, there are pre- and post-movement testing regulations, and more frequent testing is applied when a TB incident occurs, in both the infected herd and those herds considered contiguous to infected herds. In higher-risk areas, there are control methods focused on the wildlife reservoir *Meles meles*, the European badger [2,4]. 

The single intradermal comparative cervical tuberculin (SICCT) test is used in both England and Wales to establish whether an animal is a reactor (presumed infected) [8]. This uses an intradermal injection of both bovine and avian tuberculin at two adjacent sites on the neck. A positive reaction to either tuberculin is defined as an increase in skin thickness of more than 2 mm or an edematous reaction. To be deemed as a reactor, the bovine reaction must be 4 mm greater than the avian reaction under standard interpretation and 2 mm greater (or a positive bovine reaction with a negative avian reaction) under severe interpretation. An inconclusive reactor (IR) at standard interpretation is an animal with a positive bovine reaction which is greater than a positive avian reaction by less than 4 mm, or greater than a negative avian result by 4 mm or less. Classification of an IR under severe interpretation differs between England and Wales, although both work on the principle of reducing the difference between bovine and avian reactions relative to standard reactors and, therefore, the threshold at which an animal is identified as potentially infected [8]. The comparative nature of the test increases specificity by preventing the false classification of animals infected with mycobacteria other than *M. bovis* as reactors due to cross-reactivity [9].

While the specificity of the SICCT test is generally accepted to be near perfect [9,10], estimates of median sensitivity at the animal level are as low as 50% and as high as 80% [9,11]. Using the severe interpretation increases the sensitivity of the test; however, it is still possible that an infected animal may go undetected.

Inconclusive reactors are not subject to compulsory slaughter on the basis of the first IR test result unless they have a further inconclusive or positive result when re-tested, deeming them to be a reactor [8]. The policies for IR animals differ in England and Wales, with Wales taking into account whether the IR was detected under standard or severe interpretation and utilizing the interferon gamma (IFN-γ) test for re-testing [8].

Proposed reasons for infected cattle with an inconclusive or a negative SICCT test result include testing in the early stages of infection before the immune system fully responds, terminal TB infection causing anergy, and immunosuppression related to disease, drugs, and the early post-calving period [9]. Conversely, an IR animal may not be infected. Reasons for an inconclusive result in uninfected animals may include exposure to other mycobacteria or errors in test administration [11]. A study of cattle in officially TB-free herds in Ireland that had an IR result showed that the percentage confirmed as infected by post-mortem histology, with or without culture, was lower than the percentage of reactor animals and higher than that of test negative animals [12]. Similarly, epidemiological analysis of TB surveillance data from 2016 conducted by the Animal and Plant Health Agency (APHA) showed the percentage of IRs slaughtered for suspect TB that had visible lesions post mortem was less than half that observed in reactors in England [5]. Detection of lesions post mortem in IRs indicates that some infected cattle may be evading the SICCT test as currently interpreted, and there is uncertainty with regard to the threshold at which an animal should be defined as positive. 

Currently, when an IR is detected, the cattle should be isolated from the rest of the herd and restricted from moving. However, movement restrictions are not applied to the rest of the herd unless reactors were also found or there was a TB incident within the herd in the past three years [5]. Movement restrictions on IRs are lifted if an IR tests negative at re-test and the IR was detected in a low-risk area (LRA) herd with no SICCT test reactors or slaughterhouse cases of TB. Resolved IRs in the HRA, the edge area, and IRs that occurred during a TB incident involving other cattle in the LRA of England are subject to lifelong restrictions within the herd since November 2017 [8].

There is evidence that IR animals can spread the disease. IR reactor cattle in Ireland which tested negative on the re-test and were subsequently moved to a new herd within the following six months were 12 times more likely to be TB-positive at the next test [12]. Longer-term risk was also demonstrated in cohort studies in Ireland and in England and Wales. In Ireland, IRs that were negative at re-test were followed over a four-year period, and 9.3% of these animals were subsequently diagnosed with TB compared to 2.6% of control animals [13]. In Northern Ireland, in a high-risk cohort, animals with an inconclusive penultimate skin test result had an elevated adjusted odds ratio (OR) of 2.84–3.89 (*p* < 0.001) relative to non-reactors for the presence of bTB lesions at slaughter [14]. In England and Wales, IR-only herds were 2.7 times more likely to have a subsequent incident compared with negative-testing herds, although this difference reduced over time [15].

While the fate of IR cattle in England and Wales was studied at the herd level [15], it was not explored and reported at the individual animal level. Investigating the association between an animal’s IR status and the odds of them subsequently becoming a reactor, as well as other factors associated with individual animal risk, could help farmers make an informed choice about keeping them, as well as aid decision-making with regard to national control. The aim of this study was to test the hypothesis that the odds of becoming a reactor increase in IR animals compared to animals that tested negative at a previous test. 

## 2. Methods 

### 2.1. Study Population

The study population included all cattle in herds that were classified as IR-only herds at their first whole-herd test (WHT) using the SICCT test in 2012 in England and Wales. Cattle within this population that went on to be slaughtered as a result of the first WHT in 2012 were removed from the study. The study spanned a five-year period from January 2012 to December 2016. Cases were defined as animals classified as reactors during the study period after the first WHT in 2012 as a result of a positive SICCT test (this included animals slaughtered as a result of a consecutive positive IR or reactor test result in line with the differing legislation in England and Wales), a positive IFN-γ test, or lesions suspicious of TB observed during slaughterhouse surveillance. All other animals were considered controls. The exposure of interest was the animal’s test status at the first WHT in 2012 (IR or negative). 

### 2.2. Data Source 

The data were obtained from the APHA’s Sam database for TB test data and were prepared using Microsoft SQL Server 2012 and extracted for cleaning and analysis using IBM SPSS v25 and Stata v14.2 (Stata Corporation, College Station, TX, USA). A data dictionary was generated to describe the contents of each field. Each field was assessed for missing values, and only herd size (number of animals currently in the herd) was found to be incomplete. Herd size was scaled up to represent 100 animals (i.e., by dividing by 100) in order to aid interpretation of the results. String variables were re-coded into integers or Boolean values for analysis. The dataset included information on both the 2012 WHT and the subsequent TB test results, as well as individual and herd-level information for each animal. 

### 2.3. Statistical Analysis 

Descriptive analyses were carried out to assess the number and proportion of cases in the study population, the number and proportion of cattle according to IR status, and other possible explanatory variables: sex, IR results previous to the 2012 WHT, geographical risk area, herd type, first test type (e.g., routine surveillance tests, IR re-test, etc.) first test classification, number of herd incidents in the last 10 years, and herd size. 

Mixed-effects logistic regression was performed for the outcome variable (subsequent reactor result) and each explanatory variable, including herd as a random effect. Those variables potentially associated with a subsequent reactor result (*p* < 0.20) were included in the multivariable analyses. Five area-specific multivariable logit models were developed: one for England as a whole including an interaction between first WHT status in 2012 and risk area, one for Wales as a whole, and one for each of the HRA, edge area, and LRA of England. 

For each model, we started with all variables brought forward from the bivariable analysis. The order of these variables in the model was guided by the relationship between each variable and the outcome variable (i.e., size of the odds ratio). Variables were systematically removed in a backward stepwise manner starting with the variable with the largest non-statistically significant *p*-value. 

Continuous variables were assessed as log-transformed and untransformed values and as categorical variables to determine which had the most influence on the odds ratio for IR status. The continuous values had the most influence and were used in the statistical analysis. Continuous variables were also checked for linearity and multicollinearity. 

The likelihood ratio test (LRT) was used to assist model building and to compare the mixed-effects models to normal logistic regression models not accounting for clustering by farm. Model outputs included an estimate of variance between farms (*sigma*, where 0 = no variation between farms) and an estimate of correlation within farms (*rho*, where 0 = no correlation within farms). Parameter estimates were obtained using quadrature approximations [16], whose accuracy depends partially on the number of integration points used; thus, we checked the reliability of the approximations using the Stata quadchk command. This refits the model for different numbers of integration points and then compares the different solutions. Models were refitted using 16 integration points and compared to the default fitted approximation using 12 integration points by calculating the relative differences. Parameter estimates were considered to be reliable if the relative difference was less than 0.01. Influential points were examined using the leverage (hat diagonal) plotted against the predicted probabilities (Supplementary Materials Figures S1 and S2). The 5% of observations with the greatest leverage were excluded and the mixed-effects models were re-run to see if the estimates differed. A *p*-value for a parameter estimate of less than 0.05 in the final model was taken as evidence of a statistically significant association between each variable and becoming a subsequent reactor.

## 3. Results

### 3.1. Descriptive and Bivariable Analyses

The total number of cattle included in the study was 580,530. The number and percentage of animals that became a reactor in the study period, stratified by IR status at the first WHT in 2012, are summarized in Table 1. There was a greater percentage of cases among IR animals (11.2%) compared to negative animals (1.9%). Among IR animals, around 58% were classified as reactors by an IR re-test (test code VE-IR), and 50% were classified as reactors within 84 days. A value for herd size was missing for 4778 cattle.

Bivariable analysis showed that the odds of becoming a subsequent reactor were eight times greater among animals that were classified as an IR at the first WHT in 2012 compared with animals with a negative test result (*p* < 0.001) (Table 1).

Bivariable analysis of potential explanatory variables showed that the odds of becoming a subsequent reactor were greater for females compared with males, for animals in the edge and HRA of England and in Wales compared with the LRA of England, for dairy animals, and for those with an edematous or circumscribed reaction described at the 2012 WHT (Table 1). For the continuous variables (Table 2), the median number of herd incidents in the past 10 years was higher for the herds of animals which became subsequent reactors, whilst median herd size was smaller. An increase in past incidents was found to be statistically significantly associated with a subsequent reactor result (Table 2). Increasing herd size and animals with IR results recorded prior to the 2012 WHT both produced parameter estimates indicating a negative association with becoming a reactor, but neither were statistically significant.

### 3.2. Multivariable Analyses

#### 3.2.1. England Model

The LRT indicated that the model containing the interaction term between IR status and risk area was statistically significantly different from the model without the interaction (*p* = 0.043), and that area-specific models should be explored. Animals in the LRA with an inconclusive result at the 2012 WHT were 18.22 times more likely to have been confirmed as a reactor by the end of the study period than animals which had a negative test result (95% confidence interval (CI): 8.81–37.66; *p* < 0.001) (Table 3). However, the odds of becoming a reactor following an inconclusive result at the 2012 WHT were 59% lower in the edge area (OR: 0.41; 95% CI: 0.18–0.92; *p* = 0.032) and 63% lower in the HRA (OR: 0.37; 95% CI: 0.18–0.78; *p* = 0.008) compared with the LRA. Other factors associated with increased odds of becoming a reactor in England included being female, having a circumscribed lesion at the first test, and increasing numbers of incidents in the herd during the 10-year period prior to the study. 

The estimates of *sigma* and *rho* were 1.44 (95% CI: 1.37–1.50) and 0.39 (95% CI: 0.36–0.41), respectively. The LRT generated a *p*-value < 0.001, indicating strong evidence against the null hypothesis of no correlation. None of the relative differences for the quadrature comparisons were greater than 0.01, which indicated that the parameter estimates were reliable. Parameter estimates did not differ greatly when the 5% of observations with the greatest leverage were removed (Supplementary Materials Table S1).

#### 3.2.2. Wales Model

In Wales, animals with an inconclusive result at the 2012 WHT were 6.87 times more likely to have been confirmed as a reactor by the end of the study period than animals which had a negative test result (95% CI: 5.75–8.22; *p* < 0.001) (Table 4). Other factors associated with increased odds of becoming a reactor in Wales were being female, being in a dairy herd, and increasing numbers of incidents in the herd during the 10-year period prior to the study. An animal having a previous IR result recorded appeared to be significantly associated with a decrease in the odds of becoming a reactor. 

The estimates of *sigma* and *rho* were 1.54 (95% CI: 1.41–1.68) and 0.42 (95% CI: 0.38–0.46), respectively. The LRT generated a *p*-value < 0.001, indicating strong evidence against the null hypothesis of no correlation. None of the relative differences for the quadrature comparisons were greater than 0.01, which indicated that the parameter estimates were reliable. Parameter estimates did not differ greatly when the 5% of observations with the greatest leverage were removed (Supplementary Materials Table S1).

#### 3.2.3. HRA Model

The odds of an animals being confirmed as a reactor by the end of the study period were 6.85 times greater for animals with an inconclusive result at the 2012 WHT than animals which had a negative test result (95% CI: 5.98–7.86; *p* < 0.001) (Table 5). Other factors associated with increased odds of becoming a reactor in the HRA were being female, increasing numbers of incidents in the herd during the 10-year period prior to the study, and having a circumscribed lesion at the first test. Increasing herd size was significantly associated with a decrease in the odds of becoming a reactor. 

The estimates of *sigma* and *rho* were 1.38 (95% CI: 1.31–1.45) and 0.37 (95% CI: 0.34–0.39), respectively. The LRT generated a *p*-value < 0.001, indicating strong evidence against the null hypothesis of no correlation. None of the relative differences for the quadrature comparisons were greater than 0.01, which indicated that the parameter estimates were reliable. Parameter estimates did not differ greatly when the 5% of observations with the greatest leverage were removed (Supplementary Materials Table S1).

#### 3.2.4. Edge Area Model

In the edge area of England, animals with an inconclusive result at the 2012 WHT were 8.79 times more likely to have been confirmed as a reactor by the end of the study period than animals which had a negative test result (95% CI: 5.92–13.04; *p* < 0.001) (Table 5). Being female was the only other variable associated with increased odds of becoming a reactor in the edge area. The estimates of *sigma* and *rho* were 1.84 (95% CI: 1.56–2.17) and 0.51 (95% CI: 0.42–0.59), respectively. The LRT generated a *p*-value < 0.001, indicating strong evidence against the null hypothesis of no correlation. None of the relative differences for the quadrature comparisons were greater than 0.01, which indicated that the parameter estimates were reliable. Parameter estimates did not differ greatly when the 5% of observations with the greatest leverage were removed (Supplementary Materials Table S1).

#### 3.2.5. LRA Model

Animals with an inconclusive result at the 2012 WHT were 22.68 times more likely to have been confirmed as a reactor by the end of the study period than animals which had a negative test result (95% CI: 10.75–47.86; *p* < 0.001) (Table 5). No other variables were associated with the odds of becoming a reactor in the LRA. 

The estimates of *sigma* and *rho* were 1.89 (95% CI: 1.34–2.68) and 0.52 (95% CI: 0.35–0.69), respectively. The LRT generated a *p*-value < 0.001, indicating strong evidence against the null hypothesis of no correlation. None of the relative differences for the quadrature comparisons were greater than 0.01, which indicated that the parameter estimates were reliable. Parameter estimates did not differ greatly when the 5% of observations with the greatest leverage were removed (Supplementary Materials Table S1).

**Figure 1:**
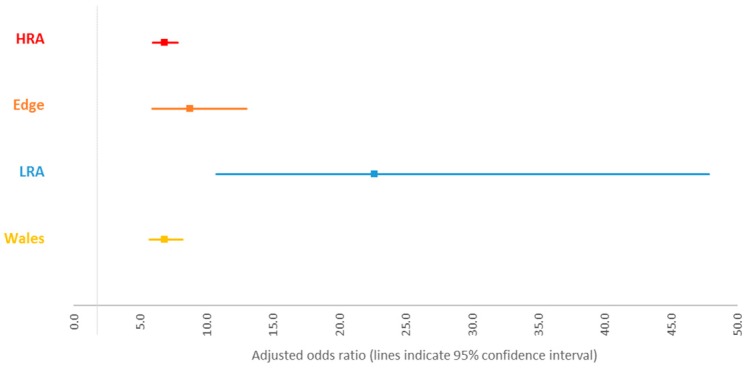
Odds of becoming a reactor for animals with an inconclusive result at the first whole-herd test (WHT) in 2012 for each area. Note: each odds ratio was derived from a separate model.

A summary of the odds of becoming a reactor based on IR status for each area of England and for Wales estimated from the area-level models is presented in Figure 1. Increased odds of becoming a reactor following an IR result were observed for all areas. The effect was similar across the HRA, edge area, and Wales, while a larger effect was observed in the LRA. The confidence interval for this estimate overlapped with the confidence interval for the estimate in the edge area, but not with the other areas (Figure 1).

## 4. Discussion

This is the first study to explore the risk posed by IR animals in England and Wales. An understanding of the importance of IR animals for infection transmission can guide control measures as England and Wales work toward TB-free status. In this study, IR animals were more likely to become reactor animals over the five-year follow-up period than animals which tested negative at a WHT at the beginning of the follow-up. This suggests that IR cattle have an increased risk of being or becoming infected with TB. Similar results were demonstrated in Irish cattle through a greater proportion of IR animals slaughtered and confirmed as TB-positive at slaughter compared to negative animals when slaughtered prior to a re-test, and through subsequent testing over a four-year period [12,13]. Similarly, in Northern Ireland, animals in a high-risk cohort with an inconclusive penultimate skin test result had an elevated adjusted OR of 2.84–3.89 (*p* < 0.001) for the presence of bTB lesions at slaughter [14].

In the present study, it was not possible due to the structure of the data to identify with certainty which IR animals were disclosed as reactors by the IR re-test rather than a test subsequent to the IR testing pathway. However, based on the type of test which ultimately disclosed the animal as a reactor, and the time between the initial IR result and becoming a reactor, we can estimate that just over one-half of IRs that go on to become reactors were classified as reactors at the re-test. It is not known whether those animals that tested clear at the re-test and went on to become reactors were infected at the time of the re-test and, thus, missed by the re-testing policy, or infected after the re-test. 

The interaction term in the England model and the area-specific models showed that the increased odds for an IR animal becoming a reactor was similar across the HRA, edge area, and Wales, while a larger effect was observed in the LRA. The confidence interval for this estimate was wide, most likely due to the smaller sample size in this area, yet it did not overlap with the estimates for the HRA and Wales. Overall incidence and prevalence of TB is much lower in the LRA than in the other TB risk areas, indicating that the absolute or background risk for TB in the LRA is much lower. The results suggest that the risk for TB in an IR animal in the LRA is much higher than in IR animals in other areas. This could suggest that the increased risk is more likely due to an intrinsic risk in the animal (e.g., it was already infected when disclosed as an IR) than subsequent exposure to *M. bovis* because of the low background prevalence in this area. It also suggests that the attributable risk for TB posed by IRs is greater in the LRA compared to other areas. However, it is important to remember that the population studied here consists of IR-only herds, and it is possible that the difference between areas might be because IRs are more likely to be found in herds that have infection in other areas.

The bivariate analysis showed that animals with a history of IR results to previous tests prior to the start date of the study had decreased odds of becoming a reactor compared to animals with no previous IR results. However, once area and other variables were accounted for in the multivariable models, previous IR results reduced the odds of becoming a reactor in the Wales model only. In Irish cattle, animals which had multiple IR results during a four-year study had a shorter time to TB diagnosis than those which only had a single IR result, for whom risk of TB diagnosis reduced 500 days post test [13]. A possible explanation for a reduced time to diagnosis with multiple IR results, as well as a factor for consideration with regard to the increased odds of becoming a reactor following any one IR result, is the increased testing that the animal undergoes. This may have had more impact in the Irish study due to the observation of multiple IR results within the study period [13]. In comparison, this study considered historical IR results, and no data were available on tests between the start of the study and a condemning result. 

The relationship of differing numbers of IR results over an animal’s lifetime and detection of TB may be worth further investigation, to determine if post-mortem diagnostics reveal a difference in the number of confirmed cases of TB in these animals compared to animals with a single IR result. One IR result could be due to errors in administering or reading the test: the administration of tuberculin in incorrect volumes, at the incorrect site, or fractious animals causing difficulties in reading the test [9,17]. This is a much less likely explanation for multiple IR results in the same animal. There could be a trend toward repeat IR animals never developing a positive reaction to the SICCT test despite TB infection due to immunomodulatory factors such as genetics or pre-exposure to *Mycobacterium avium* [18,19,20]. Amos et al. (2013) investigated a possible effect of a particular TB genotype, which was noted to be prevalent in non-reactor animals. Increases in skin thickness were lower in animals with the “22” genotype [21], although a subsequent study investigating the same genomic region in a larger dataset did not find any evidence to support these findings [22]. It was also observed that the difference in skin thickness between the bovine tuberculin site and avian tuberculin site was reduced in animals pre-exposed to *Mycobacterium avium* [23].

With regard to other TB risk factors, the negative relationship between herd size and becoming a subsequent reactor observed in the HRA was unexpected. Increasing herd size is widely accepted as a risk factor for occurrence of a TB incident [19,24]. Here, however, it appeared that an increase in herd size reduced the odds of becoming a reactor in this risk area, although the effect was small. The difference here could be related to the sensitivity of the SICCT improving with the number of animals tested, i.e., it is better at detecting disease on a herd basis than an individual basis [9,25]. It may be that herd size is a risk factor for a TB incident at the herd level due to the increased chance of detecting a positive animal amongst the group; however, this is unlikely to affect the probability of an individual animal being detected by the test. 

Female IRs were found to have greater odds of becoming a reactor compared with males in all areas except the LRA. This could be due to increased longevity of female animals for breeding and milk production in England and Wales. Interestingly, the effect of sex was not removed by inclusion of herd type in the model. IRs in dairy herds were significantly more likely to become a reactor in the Wales model although not in the models for other areas. From a herd level point of view, there is conflicting evidence on the association between herd type and TB risk. Epidemiological analysis showed dairy herds are 9% more likely to have a TB incident than beef herds in England after adjustment for location and herd size [5]. Another study examining risk factors associated with the hazard of a TB incident found no association for herd type, although there was evidence that the hazard of a TB incident among dairy herds increases over time [15]. On an individual animal level, the evidence is limited and variable. Downs et al. found that dairy cattle in Great Britain (GB) with a positive SICCT test result were less likely to have *M. bovis* infection confirmed post mortem than other cattle subject to similar TB testing regimes, which might indicate a greater sensitivity of the SICCT test in dairy breeds [26]. The mean difference between avian and bovine reaction size was greater in dairy animals than non-dairy confirmed reactors in Northern Ireland [27]. If the difference between the reactions is typically always greater in infected dairy animals, fewer would be missed as false negatives or classified as an IR in comparison to beef animals, which might affect long-term probabilities of being disclosed as a reactor. However, Amos et al. (2013), including clear, IR, and reactor animals, found smaller reaction sizes to both bovine and avian tuberculin in common dairy breeds compared to beef breeds [21]. As our study does not include assessment of bovine and avian skin test reactions nor reactor animals, it is not possible to compare findings. 

TB history (an increasing number of incidents in the herd over the previous 10 years) was significantly associated with the odds of becoming a reactor in the England, HRA, and Wales models, and this correlates with the findings from herd-level studies and the positive association between the positive predictive value of tests with background prevalence of infection [15,28,29]. No association between TB history and the odds of becoming a reactor was observed in the edge area or LRA. 

The risk factors included as potential confounders in this study were selected based on evidence in the literature that they increase the risk of TB, and they were available from the surveillance database. Although important risk factors were included in the analysis, there are also a large number of risk factors for which there were no available data, particularly at the animal level. Over 41 risk factors of varying importance have been reported in the literature [28], and there are likely to be others as yet identified [19]. 

The frequency of testing would have differed throughout the study for each animal according to their location and test history. The study also does not take into account time alive following the WHT of 2012. This could introduce bias in a couple of ways. Firstly, animals that live for longer through the study period could have been subject to a greater number of tests and, therefore, could have had greater opportunity to be confirmed as a reactor. Secondly, the number of IR animals that become reactors may have been underestimated, as it is possible that IR animals were slaughtered sooner than negative animals following the WHT due to the anticipated risks associated with them. Studies demonstrated farmer concern over IR animals, with support for the isolation of IR animals and observed shorter time to slaughter than for negative animals in the Republic of Ireland and Northern Ireland [13,30]. It was hypothesized that an IR result could be due to immunosuppression [18], and it is, therefore, plausible that the health of these animals is compromised, resulting in a higher death rate compared to animals with negative SICCT tests. This is an area that requires further investigation. 

The study demonstrated that animals from IR-only herds, with an IR test result, were more likely to become a reactor during the study period than animals with a negative result at the same test. This effect was found in all TB risk areas in England and in Wales, but the odds were significantly higher in the LRA. Currently, we have insufficient information with which to infer the causal pathway between detection as an IR and detection as a reactor and to understand difference between areas. This study does, however, support the need to consider differential controls of these animals in different risk areas since the proportion of disease attributable to these animals is likely to differ between areas. The study demonstrated that the effects of some of the traditionally accepted risk factors for TB are important on an individual animal basis, as well as a herd level basis, such as sex and TB history of the herd, but that the importance of some of these factors differs according to area. This highlights the importance of area-specific policies to tackle TB. This study also highlighted some factors that warrant further investigation on an individual animal level such as herd type, multiple IR results, and herd size. These findings can help inform decision-making in relation to the management of IR animals, both at national and farm level.

## Figures and Tables

**Table 1:** Number and percentage of cattle which went on to become a subsequent reactor according to each explanatory categorical variable, and the association between each variable and subsequent reactor status. WHT—whole-herd test; IR—inconclusive reactor; LRA—low-risk area; HRA—high-risk area.

Variable		*N*	Subsequent Reactor				OR ^1^	95% CI ^2^		*p*-Value
			No		Yes					
			*N*	%	*N*	%				
First WHT status in 2012										
	Negative	573,309	562,654	98.1	10,655	1.9	1.00			
	IR	7221	6414	88.8	807	11.2	8.09	7.42	8.82	<0.001
Sex										
	Male	100,931	100,082	99.2	849	0.8	1.00			
	Female	479,599	468,986	97.8	10,613	2.2	2.40	2.22	2.59	<0.001
IR result previous to 2012 WHT										
	No	567,021	555,876	98.0	11,145	2.0	1.00			
	Yes	13,509	13,192	97.7	317	2.3	0.98	0.87	1.10	0.740
Risk area										
	England—LRA	22,339	22,270	99.7	69	0.3	1.00			
	England—Edge	48,155	47,466	98.6	689	1.4	4.20	2.50	7.04	<0.001
	England—HRA	370,694	362,242	97.7	8452	2.3	10.78	6.80	17.08	<0.001
	Wales	139,342	137,090	98.4	2252	1.6	6.14	3.83	9.82	<0.001
Herd type										
	Beef	206,022	202,490	98.3	3532	1.7	1.00			
	Dairy	373,590	365,670	97.9	7920	2.1	1.29	1.13	1.47	<0.001
	Other	918	908	98.9	10	1.1	1.85	0.50	6.80	0.355
First test classification ^3^										
	No lesion	501,777	492,451	98.1	9326	1.9	1.00			
	No edema	30,820	30,287	98.3	533	1.7	1.07	0.96	1.18	0.228
	Some edema	4463	4295	96.2	168	3.8	2.77	2.32	3.31	<0.001
	Circumscribed	43,470	42,035	96.7	1435	3.3	2.12	1.99	2.26	<0.001

^1^ Odds ratio rounded to two decimal places. ^2^ Confidence interval rounded to two decimal places. ^3^ First test classification relates to the observed reaction at the site of injection of tuberculin.

**Table 2:** Summary of continuous explanatory variables according to whether or not the cattle became a subsequent reactor, and the association between each variable and subsequent reactor status.

Variable	*N*	Subsequent Reactor				OR ^1^	95% CI ^2^		*p*-Value
		No		Yes					
		Median	Range	Median	Range				
Number of herd incidents in the last 10 years.	580,530	1	0–8	2	0–8	1.26	1.21	1.31	<0.001
Herd Size ^3^	575,752	287	0–3029	257	0–2817	0.98	0.95	1.00	0.065

^1^ Odds ratio rounded to two decimal places. ^2^ Confidence interval rounded to two decimal places. ^3^ Scaled herd size values used to calculate OR.

**Table 3:** Multivariable logistic regression model for variables associated with becoming a subsequent reactor in England, *n* = 437,609.

Variable		OR ^1^	95% CI ^2^		*p*-Value
Status at first WHT in 2012					
	Negative	1.00			
	IR	18.22 ^3^	8.81	37.66	<0.001
Sex					
	Male	1.00			
	Female	2.25	2.06	2.45	<0.001
IR result previous to 2012 WHT					
	No	1.00			
	Yes	0.85	0.75	0.98	0.002
Risk area					
	England—LRA	1.00			<0.001
	England—Edge	4.95 ^4^	2.90	8.42	<0.001
	England—HRA	11.34 ^4^	6.99	18.40	<0.001
First test classification					
	No lesion	1.00			
	No edema	1.04	0.93	1.17	0.490
	Some edema	1.13	0.93	1.39	0.225
	Circumscribed	1.19	1.09	1.31	<0.001
Herd breakdowns in the last 10 years		1.14	1.09	1.19	<0.001
Interaction between IR status and risk area					
	England—LRA	1.00			
	England—Edge	0.41 ^5^	0.18	0.92	0.032
	England—HRA	0.37 ^5^	0.18	0.78	0.008

^1^ Odds ratio rounded to two decimal places where possible. ^2^ Confidence interval rounded to two decimal places. ^3^ Effect of IR status on becoming a reactor in the LRA. ^4^ Effect of risk area on becoming a reactor when IR status is negative. ^5^ Difference in effect when IR status is positive.

**Table 4:** Multivariable logistic regression model for variables associated with becoming a subsequent reactor in Wales, *n* = 138,143.

Variable		OR ^1^	95% CI ^2^		*p*-Value
Status at first WHT in 2012					
	Negative	1.00			
	IR	6.87	5.75	8.22	<0.001
Sex					
	Male	1.00			
	Female	2.36	1.99	2.81	<0.001
IR result previous to 2012 WHT					
	No	1.00			
	Yes	0.59	0.46	0.75	<0.001
Herd type					
	Beef	1.00			<0.001
	Dairy	1.55	1.18	2.03	<0.001
	Other	0.32	0.01	8.41	0.497
Herd breakdowns in the last 10 years		1.34	1.22	1.48	<0.001

^1^ Odds ratio rounded to two decimal places where possible. ^2^ Confidence interval rounded to two decimal places.

**Table 5:** Multivariable logistic regression models for variables associated with becoming a subsequent reactor in the HRA, edge area, and LRA of England.

**HRA**	**Variable**	**OR ^1^**	**95% CI ^2^**		***p*-Value**
	Status at first WHT in 2012				
	Negative	1.00			
	IR	6.85	5.98	7.86	<0.001
	Sex				
	Male	1.00			
	Female	2.25	2.06	2.45	<0.001
	First test classification				
	No lesion	1.00			
	No edema	1.04	0.91	1.17	0.587
	Some edema	1.12	0.91	1.37	0.306
	Circumscribed	1.17	1.06	1.29	0.002
	Herd breakdowns in the last 10 years	1.16	1.10	1.21	<0.001
	Number of cattle in herd (per 100 cattle)	0.95	0.92	0.98	0.002
**Edge**	**Variable**	**OR ^1^**	**95% CI ^2^**		***p*-Value**
	Status at first WHT in 2012				
	Negative	1.00			
	IR	8.79	5.92	13.04	<0.001
	Sex				
	Male	1.00			
	Female	2.76	1.73	4.38	<0.001
**LRA**	**Variable**	**OR ^1^**	**95% CI ^2^**		***p*-Value**
	Status at first WHT in 2012				
	Negative	1.00			
	IR	22.68	10.75	47.86	<0.001

^1^ Odds ratio rounded to two decimal places where possible. ^2^ Confidence interval rounded to two decimal places.

## Supplementary Materials

The following are available online at https://www.mdpi.com/2306-7381/6/4/97/s1: Figure S1: Plot of leverage against predicted probabilities for full model; Figure S2: Plot of leverage against predicted probabilities for full model, with 5% of observations with greatest leverage excluded; Table S1: Model outputs with 5% of observations with greatest leverage excluded.
