# Supplementary material for: Exploring the Risk Posed by Animals with an Inconclusive Reaction to the Bovine Tuberculosis Skin Test in England and Wales
Source: Vet Sci. 2019 Nov 30;6(4):97. doi: 10.3390/vetsci6040097 (PMC6958475; doi:10.3390/vetsci6040097)
Supplement: Supplementary file 1 [file vetsci-06-00097-s001.pdf]

Supplementary Information

Exploring the risk posed by animals with an inconclusive reaction to the bovine tuberculosis skin test in England and Wales

Elizabeth May, Alison Prosser, Sara H. Downs and Lucy A. Brunton

Influential points were examined using the leverage (hat diagonal) plotted against the predicted probabilities. The 5% of observations with the greatest leverage were excluded and the mixed effects models re-run to see if the estimates differed.

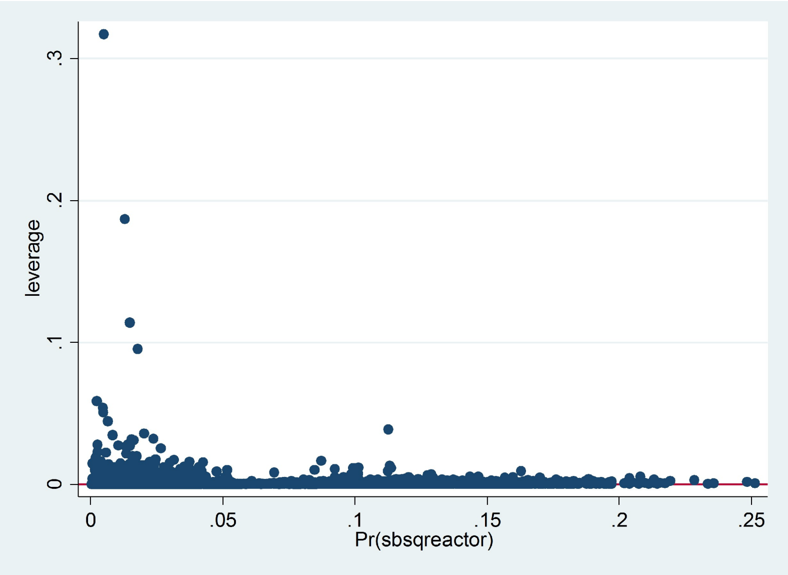

Figure S1. Plot of leverage against predicted probabilities for full model.

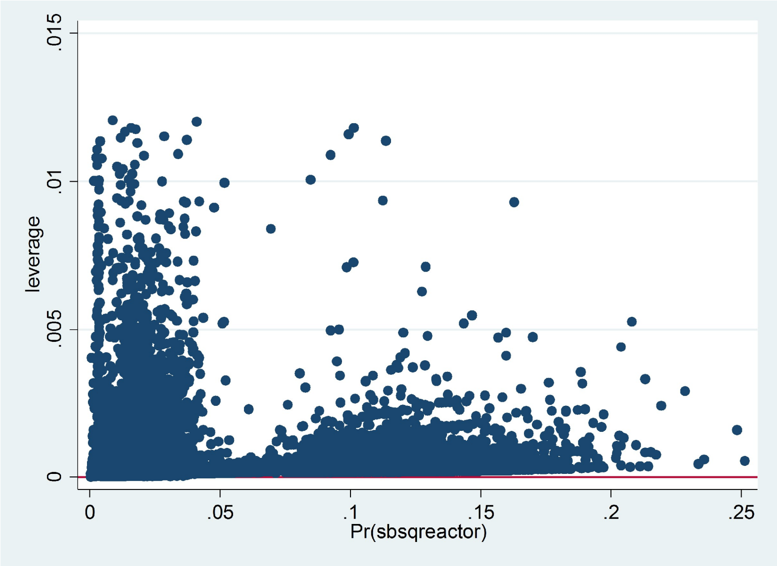

Figure S2. Plot of leverage against predicted probabilities for full model, with 5% of observations with greatest leverage excluded.

Table S1. Model outputs with 5% of observations with greatest leverage excluded.

England

**Variable**		**OR**	**[95% Conf.**	**Interval]**	**P value**
Status at first WHT in 2012	Negative	1.00			
	IR	21.70	10.14	46.45	<0.001
Risk area	LRA	1.00			
	Edge	5.69	3.20	10.14	<0.001
	HRA	13.00	7.64	22.14	<0.001
First test classification	No lesion	1.00			
	No oedema	1.05	0.93	1.18	0.450
	Some oedema	1.16	0.94	1.43	0.157
	Circumscribed	1.20	1.09	1.31	<0.001
Sex	Male	1.00			
	Female	2.24	2.06	2.45	<0.001
Herd breakdowns in the last 10 years		1.14	1.08	1.19	<0.001
Interaction between IR status and risk area					
	England -LRA	1.00			
	England - Edge	0.34	0.15	0.80	0.013
	England - HRA	0.31	0.14	0.67	0.003

HRA

**Variable**		**OR**	**[95% Conf.**	**Interval]**	**P value**
Status at first WHT in 2012	Negative	1.00			
	IR	6.78	5.91	7.77	<0.001
First test classification	No lesion	1.00			
	No oedema	1.03	0.91	1.17	0.638
	Some oedema	1.14	0.92	1.41	0.217
	Circumscribed	1.18	1.07	1.30	0.001
Sex	Male	1.00			
	Female	2.24	2.05	2.45	<0.001
Herd breakdowns in the last 10 years		1.15	1.10	1.21	<0.001
Number of cattle in herd (per 100 cattle)		0.94	0.91	0.98	0.001

Edge

**Variable**		**OR**	**[95% Conf.**	**Interval]**	**P value**
Status at first WHT in 2012	Negative	1.00			
	IR	8.75	5.89	12.99	<0.001
Sex	Male	1.00			
	Female	2.86	1.78	4.58	<0.001

LRA

Variable		OR	[95% Conf.	Interval]	P value
Status at first WHT in 2012	Negative	1.00			
	IR	28.53	12.89	63.11	<0.001

Wales

Variable		OR	[95% Conf.	Interval]	P>z
Status at first WHT in 2012	Negative	1.00			
	IR	6.93	5.79	8.30	<0.001
Sex	Male	1.00			
	Female	2.36	1.98	2.80	<0.001
IR result previous to 2012 WHT	No	1.00			
	Yes	0.57	0.45	0.74	<0.001
Herd type	Beef	1.00			
	Dairy	1.57	1.19	2.07	0.001
	Other	1.00			
Herd breakdowns in the last 10 years		1.34	1.22	1.47	<0.001
